# Male Barroom Aggression among Members of the Australian Construction Industry: Associations with Heavy Episodic Drinking, Trait Variables and Masculinity Factors

**DOI:** 10.3390/ijerph18136769

**Published:** 2021-06-24

**Authors:** Steven Litherland, Peter Miller, Nic Droste, Kathryn Graham

**Affiliations:** 1 School of Psychology, Deakin University, Geelong 3220, Australia; 2 Centre for Addiction and Mental Health, University of Toronto, London, ON N6G 4X8, Canada; 3 National Drug Research Institute, Curtin University, Perth 6845, Australia; 4 Menzies School of Health Research, Charles Darwin University, Darwin 0810, Australia

**Keywords:** male barroom aggression, heavy episodic drinking, construction workers, trait aggression, narcissism, impulsivity, male honour, conformity

## Abstract

**Introduction and Aims:** Past research indicates heavy episodic drinking (HED), trait aggression, male honour and conformity to masculine norms are risk factors for male barroom aggression (MBA) perpetration. However, little is known about the impact of these variables on experiences of MBA victimization. Further, data derived previously, particularly in relation to perpetration have come from relatively low-risk samples comprising university students, limiting the generalizability of findings to other, at-risk male groups. Thus, the present study assessed the impact of the aforementioned variables as well as personality constructs of impulsivity and narcissism on both the perpetration of and victimization from MBA among a high-risk sample sourced from male members of the Australian construction industry. **Method:** A purposive sample of Australian male construction workers aged 18 to 69 years (*n* = 476, M_age_ = 25.90, SD_age_ = 9.44) completed individual interviews at their current place of employment or while training at various trade schools in Geelong and Melbourne, Victoria, Australia. Items related to past month HED, past year experiences of verbal and physical MBA (perpetration and victimization), trait aggression’s four factors (physical, verbal, anger, hostility), impulsivity, narcissism, male honour and conformity to masculine norms. **Results:** Participants reported high levels of verbal (24.2%) and physical (21%) MBA perpetration and verbal (33.6%) and physical (31.1%) MBA victimization. Hierarchical binary logistic regression analyses identified HED as the strongest predictor of aggression involvement, while trait physical aggression, trait anger, narcissism and conformity to norms endorsing violence and a need to win were significantly and positively associated with MBA perpetration. **Conclusions:** The present study reinforces the key relationships between heavy drinking and aspects of personality and MBA, while also highlighting narcissism as a risk factor for barroom aggression perpetration. Indeed, personality profiles and HED appear to exert stronger influences on MBA perpetration than socially constructed masculinity factors, most of which were unrelated to aggression involvement in bars, clubs or pubs.

## 1. Introduction

The association between alcohol and aggression is well established [1]. Of particular concern is violence in or around licensed venues, typically involving young men who are often under the influence of alcohol [2,3]. Barroom aggression can be defined as verbally and or physically aggressive acts inside or within close proximity to hotels, bars and nightclubs [1]. Heavy episodic drinking (HED) has long been associated with incidents of male barroom aggression (MBA). However, the consumption of alcohol in isolation does not fully explain MBA as experimental research on the relationship between alcohol consumption and aggressive behaviour generally yields medium effect sizes [4], suggesting that alcohol is a meaningful but neither a necessary nor sufficient contributor to subsequent aggression [5]. Thus, it appears that acute alcohol intoxication facilitates aggression in some but not all people, and that other personal, cultural and environmental variables play an important role [6,7].

Previous research has identified a number of key correlates shown to increase the risks of MBA, among them HED [8], trait aggression [9], aspects of male honour [8,10,11] and conformity to masculine norms [9,12]. Two other potential variables of interest are impulsivity, which has been associated with alcohol-enhanced aggression in controlled experimental conditions [13] and in real-world settings [14,15,16], and narcissism given that it is related to both problematic alcohol use [17] and aggression perpetration [18]. Thus, the present study represents the first known examination of the predictive utility of all the aforementioned variables on MBA perpetration and victimization among a high-risk sample comprising members of the Australian construction industry.

The General Aggression Model (GAM) [19] is the main theoretical framework informing the present study. The GAM provides a three-stage process in which personal and situational ‘inputs’ are dealt with by an individual’s present internal state (cognitions, level of arousal, affect and intoxication) and result in decision-making processes and behavioural outcomes [19,20]. In turn, this can influence subsequent cycles of behaviour, producing an escalation of risky actions, which can be especially true with respect to both consumption and aggression [19]. Key to the GAM’s capacity to aid our understanding of MBA is the emphasis on the ‘person in the situation’ and the likely influence of trait variables such as dispositional aggression, while also allowing scope to examine other potentially related aspects of personality such as impulsivity and narcissism in the investigation of ‘inputs’ which may serve as possible conduits between alcohol use and aggressive behaviour in or around licensed venues [19]. Further, the GAM also promotes the likely influence of other potentially important variables that can inspire aggressive behavioural outcomes such as the attitudes, values and beliefs of an individual that typically precede any involvement in a conflict scenario [19], including those associated with gender such as masculinity and the establishment and preservation of one’s male honour.

## 2. HED and MBA

While it is widely acknowledged there is no one-to-one relationship between alcohol use and human behaviour [21], a substantial body of empirical literature has shown an association between intoxication and subsequent aggression [4]. Population studies indicate that as alcohol consumption increases, so too does violence [22], especially in regions where the prevailing pattern is ‘drinking to get drunk’ [1,23]. Experimental studies have consistently found that participants were more aggressive when intoxicated [24,25] with a higher ‘blood alcohol concentration’ (BAC) increasing aggressive behaviour [26,27]. HED, in particular, has been shown to contribute to aggression within a barroom context [28,29,30,31], including an elevated risk of victimization [29].

## 3. The Association of Trait Aggression, Impulsivity, Narcissism with MBA

Alcohol’s influence is thought most potent in those already predisposed to aggressiveness [6], with trait aggression defined as an individual’s predisposition to engage in verbal and physical aggression, to hold hostile cognitions and to experience and express anger [32]. When measured as a unitary construct, trait aggression has been shown to increase the risks of alcohol-related aggression (ARA) generally [25] and barroom aggression perpetration specifically [33]. When examined as unique factors, Trait Anger [34], Trait Hostility [35], Trait Verbal [24] and Trait Physical [24] have all been associated with intoxicated aggression, with the latter subscale also linked with aggression severity in bars [36]. Less is known, however, about their respective association with MBA victimization. A previous study used the ‘Trait Physical’ scale in comparisons between MBA perpetrators, victims and those reporting no MBA aggression involvement and found that those reporting perpetration scored higher than victims on Trait Physical while no significant differences were found between victims and those citing no involvement in MBA [31].

Personality traits other than trait aggression, such as impulsivity and narcissism, may also serve as potential conduits between alcohol use and subsequent aggression, particularly in barroom contexts. Impulsivity can be conceptualized broadly as a tendency to act without thinking of the consequences, to take risks in the pursuit of excitement and or new and novel experiences, an inability to control one’s behaviour [37], poor response inhibition, reduced ability to regulate one’s emotions (both positive and negative) and a diminished capacity to delay gratification [38]. Findings with respect to real-world aggression are equivocal with Magid et al. [39] reporting associations between impulsivity and alcohol-related problems including getting into fights, acting badly and doing mean things among a university student sample. However, Leonard and colleagues [40] found that impulsivity was not significantly related to the occurrence of barroom aggression generally, but was associated with participant reports of being hurt or injured during a violent episode. This suggests that it may not contribute consistently to incidents of aggression in public, but exert an influence on the severity of violence experienced during any interpersonal conflicts. These results require clarification among high-risk populations.

Similarly, narcissism may be of interest in MBA research given the proneness to grandiosity and hyper-sensitivity to rejection and perceived criticism may be highly significant in barroom settings where aggression is often motivated by identity threat [41,42], especially for men and when under the influence of alcohol. Narcissism has been associated with problematic alcohol use [17] and aggression [43,44]. This personality variable is a member of the so-called ‘Dark Triad’ alongside psychopathy and Machiavellianism and can be defined as having a pervasive sense of grandiosity as well as a high degree of self-absorption, favourable self-assessments and an over-inflated self-concept [17,43]. However, while narcissism is associated with perceptions of superiority, a sense of entitlement and low empathy, it can be accompanied by a vulnerability given narcissists can often have fragile views of the self that is contingent on external validation, and, thus, favourable self-concepts can be easily punctured by others who challenge or threaten their inherent egotism [17]. The “threatened egotism hypothesis” postulates that if a narcissist’s favourable self-concept is contradicted, impugned or jeopardized by others, they are likely to aggress against the source(s) of that threat [45]. However, provocation is not always a necessary prerequisite for aggression, particularly if narcissistic individuals lack self-control and an ability to regulate their emotions, deficiencies which are most striking in stressful scenarios that induce problematic reactions to their environments [45]. These deficiencies may be most profound in public drinking establishments given the many potential threats to self-esteem via the superficial judgements of others.

## 4. Male Honour, Conformity to Masculine Norms and MBA

Broadly, ‘masculinity’ reflects the endorsement and internalisation of culturally defined beliefs and standards scholars propose are important to men [46]. Masculine norms are usually operationalised as reflecting hegemonic or hyper-masculine ideologies endorsing male privilege, power and authority as well as patriarchal dominance [47,48], with men conforming to such an ideology thought to adhere to norms related to physical toughness, being competitive, maintaining emotional control, taking risks and predatory heterosexuality while condoning anti-homosexuality [47]. The barroom context is one where conformity to traditional male ideologies may be especially acute [11,49], particularly given the importance placed on gaining the approval of peers and the competition typically between young men vying for the attention of females, factors which can fuel an environment conducive to hyper-masculine behaviours, including male violence [8]. Studies assessing the influence of adherence to masculine norms on alcohol-related aggression, typically using items or subscales from the ‘Conformity to Masculine Norms Inventory’ (CMNI) [46] or its various derivatives are equivocal. Surveys of American [50] and Canadian [51] samples found that CMNI Risk-taking and CMNI Playboy (seeking multiple sexual partners) dimensions were positively associated with negative drinking consequences, including the perpetration of aggression. Endorsement of these norms also predicted MBA perpetration in a high-risk sample of Australian tradesmen, as did adherence to the CMNI Winning (importance of victory) dimension [9]. Surprisingly, this same study reported the Violence norm reduced the odds of perpetrating verbal MBA and was not significantly associated with physical MBA perpetration [9] with this latter result contrasting those of Wells et al. [51]. In related studies, conformity to the norms of Emotional Control and Heterosexual Self-presentation (HSP); importance placed on appearing heterosexual and the avoidance of appearing homosexual) were either unrelated [9] or negatively related [12] to barroom aggression perpetration. These findings indicate that the endorsement of norms is associated with violence, a desire to win, willingness to take risks and promiscuity and may influence MBA perpetration, with any relationship to victimization yet to be established.

Power displays between men are often paramount in barroom settings [8] with male-to-male aggression often condoned if done to right a perceived wrong or perpetrated in the defence of a friend or oneself [52]. Thus, issues of ‘male honour’ which incorporates ‘macho’ concerns related to a sense of masculinity and the protection of one’s or another’s social honour often involves aspects of face saving or impression management, particularly in the face of a real or perceived insult and can be especially heightened in bars, clubs or pubs [10,53]. Studies have drawn links between the defence or protection of social honour and involvement in barroom aggression [8,10].

## 5. Research Rationale

To determine the extent to which HED, trait aggression’s four factors, impulsivity, narcissism, male honour and conformity to masculine norms influence MBA involvement, both in relation to perpetration and victimization, it is imperative to study high-risk populations, where their respective influence is likely to be most profound. Members of the Australian construction industry represent such a sub-group. Construction is the fifth largest industry in Australia and is male dominated, with men accounting for close to 90% of those employed in the sector [54]. Construction sites are thought to have strong masculine cultures, where heavy drinking is a “rite of passage” for many, with members having reported high levels of alcohol use compared to other employment sectors, with alcohol consumption positively associated with acts of aggression [55]. Similarly, a sample of Australian tradesmen, many of whom were involved in the building industry, reported high rates of verbal and physical MBA perpetration compared to other populations [9]. Additionally, reinforcing their high-risk status, findings from a recent Australian study indicated that male blue-collar workers (including those in the construction industry) were more than twice as likely to be involved in barroom aggression compared to male professionals [56]. The present study represents the first known examination of the relationships between HED, trait aggression, impulsivity, narcissism, male honour and conformity to masculine norms and the perpetration of and victimization from MBA among an at-risk sample of Australian construction workers.

## 6. Research Aims and Hypotheses

This study sought to examine the predictive utility of these variables on MBA involvement (perpetration and victimization) among male construction workers in Australia. It was hypothesized that any HED (H1), trait aggression’s four-dimensions (Physical, Verbal, Anger, Hostility; H2), impulsivity (H3), narcissism (H4), male honour (H5) and conformity to certain masculine norms (Violence, Winning, Risk-taking and Playboy; H6) would be associated with increased risk of MBA perpetration while greater conformity to other masculine norms (Emotional Control, Heterosexual Self-presentation; H7) would be protective against MBA perpetration. Additionally, HED (H8) would be significantly associated with MBA victimization.

## 7. Method

### 7.1. Participants

Participants were a purposive sample of 476 male members of the Australian construction industry working or training in Melbourne and greater Geelong, Victoria, Australia, aged 18–69 years (M = 25.90, SD = 9.44). Sixteen different occupational groups recognized as part of the Australian construction industry were represented, including plumbers (*n* = 101, 21.2%), bricklayers (*n* = 100, 21%), carpenters (*n* = 92, 19.3%), painters (*n* = 29, 6.1%), electricians (*n* = 22, 4.6%), cabinet makers (*n* = 19, 4%), labourers (*n* = 19,4%), concreters (*n* = 11, 2.3%) and metal fabricators (*n* = 10, 2.1%). Ninety five percent of the people approached agreed to do an interview.

### 7.2. Procedure

Ethics approval was obtained from the Deakin University Human Ethics Advisory Group-Health (DUHREC_2013-104). The cross-sectional study design employed a face-to-face individual questionnaire, delivered by trained interviewers (2 males and 4 females). Interviews were conducted at several Technical and Further Education (TAFE) colleges and various construction sites across Melbourne and greater Geelong, Victoria, Australia. Approval from managerial, on-site supervisory or teaching staff was granted before entering each of the respective locations. Possible participants were then approached and asked if they would volunteer to participate in a study of male barroom aggression among Australian construction workers. Those who agreed were then interviewed. Data collection occurred between June and November 2016.

After providing verbal and written consent, participants were interviewed one on one by a researcher, who asked the questionnaire items orally and recorded responses either on hard copies of the entire survey or electronically using TAP Form’s software. Interviews lasted approximately 20 min. No incentives were offered to encourage participation.

## 8. Measures

The 59-item questionnaire was designed to be brief with the aim of fast and efficient delivery [2,57]. It assessed demographics in age and occupation, as well as alcohol and illicit drug use, experiences of barroom aggression and items related to trait aggression, narcissism, impulsivity, conformity to masculine norms and male honour. The present study focused on the extent to which HED, personality variables (Trait Physical, Trait Verbal, Trait Anger, Trait Hostility, impulsivity, narcissism) and issues related to masculinity (masculine norms, male honour) were associated with involvement in verbal and physical MBA perpetration and victimization. In the following, we describe measures used in the present analyses.

**Perpetration of and Victimization from MBA.** Participants reported the number of times they had been involved in an aggressive incident at a bar, club or pub during the previous 12 months with items related to physical perpetration (where the respondent grabbed, pushed, shoved, hit or kicked someone), verbal perpetration (where the respondent insulted, yelled or shouted at someone), physical victimization (where the respondent had been grabbed, pushed, shoved, hit or kicked by someone else) and verbal victimization (where the respondent had been insulted, yelled or shouted at by someone else). Items were drawn from previous research by Wells et al. [52,58]. Responses to these four forms of MBA were converted into dichotomous variables, reflecting perpetration of and victimization from verbal and physical aggression at a bar, club or pub at least once during the previous year (0 = no, 1 = yes).

**Heavy episodic drinking (HED).** Participants reported the number of times they had consumed 8–14 and 15 or more standard alcoholic drinks on a single occasion during the past month. Items were combined to form one dichotomous variable reflecting at least one heavy episodic drinking (8 or more standard alcoholic drinks—80 g of alcohol) session during the previous month versus none (0 = no, 1 = yes).

**Trait Aggression.** The short Aggression Questionnaire (AQ-12) [59] measured trait aggression’s four-factors in Trait Physical (“There are people who pushed me so far that we came to blows”), Trait Verbal (“I cannot help getting into arguments when people disagree with me”), Trait Anger (“Sometimes I fly off the handle for no good reason”) and Trait Hostility (“At times I feel I have gotten a raw deal out of life”). Participants rated the extent to which each item described them on a Likert-type scale (1 = extremely uncharacteristic of me, 3 = neutral, 5 = extremely characteristic of me), with scores on each of the three items per subscale collated and averaged. The AQ-12 has comparable construct validity to the original AQ-29 [60] and good internal reliability [59].

**Narcissism.** Narcissism was assessed using the Single Item Narcissism Scale (SINS) [44]. Participants were asked “To what extent do you agree with this statement: I am a narcissist”. (Note: The word ‘narcissist’ means egotistical, self-focused, and vain) with responses on a 7-point Likert-type scale (1 = very untrue, 4 = neither untrue or true, 7 = very true). The SINS correlates positively with several narcissism measures and is related to both grandiose and vulnerable aspects of the construct [44]. The SINS has demonstrated convergent and criterion validity as well as high test–retest reliability and is considered a suitable item when length of an overall survey is an issue for administrators [44]. The SINS, however, primarily taps into more fragile and less desirable components of narcissism [44] which may be noteworthy when examining predictors of MBA.

**Impulsivity.** This construct was assessed via three items used previously in alcohol and aggression research, including among construction workers in the U.S. [61]. Participants were asked how well the following statements described them: (1) I often act on the spur-of-the-moment; (2) You might say I act impulsively; (3) Many of my actions seem to be hasty. Responses spanned a 4-point Likert-type scale (1 = Not at all, 2 = A little, 3 = Some, 4 = Quite a lot).

**Male Honour.** This was examined using two items derived from Wells et al. [52] ‘Belief and Attitudes toward Male Alcohol-Related Aggression’ (BAMARA) Inventory. Participants were asked to indicate the extent to which they (dis)agree with the following statements: “Guys are cowards if they back down from a fight at a bar” and “I’d be ashamed of myself if I didn’t stand up to a guy who was threatening to fight me at a bar”. Responses were on a 5-point Liket-type scale (1 = strongly disagree, 3 = neither, 5 = strongly agree). Elements of the BAMARA Inventory have been used reliably in previous studies [8,62].

**Conformity to Masculine Norms.** Eighteen items from the nine-factor CMNI-46 [63] were used, with three items across each of the six subscales chosen for inclusion in the present study [9]: CMNI Winning (“It is important for me to win”), CMNI Violence (“Sometimes violent action is necessary”), CMNI Emotional Control (“I tend to keep my feelings to myself”), CMNI Risk-taking (“I enjoy taking risks”), CMNI Playboy (“I would feel good if I had many sexual partners”), CMNI Heterosexual Self-presentation (“It would be awful if people thought I was gay”). The CMNI-46 is widely used [50,51] and its subscales have good construct validity and internal reliability. Participants rated the extent to which each of the items described their own actions, feelings and beliefs on a 4-point Likert-type scale (1 = strongly disagree, 4 = strongly agree). Responses to each of the three items per subscale were collated, then averaged.

## 9. Analyses

First, descriptive statistics including means, standard deviations and correlations between key variables were computed. Next, four hierarchical binary logistic regression analyses were conducted to determine the extent to which HED, personality variables in trait aggression’s four-factors (Physical, Verbal, Anger and Hostility), impulsivity, and narcissism as well as six masculine norms subscales (Winning, Violence, Emotional Control, Risk-taking, Playboy, Heterosexual Self-presentation) and male honour contributed to the perpetration of and victimization from physical and verbal MBA. In each model, at step 1, the personality traits were entered to gauge their effects after controlling for HED while masculine norms and male honour were entered at step 2 in order look at the influence of socially constructed masculinity factors after controlling for HED and personality traits which were expected to be more profound contributors to MBA. Analyses were conducted using statistical software package IBM SPSS Statistics 22. Regression analyses were run including and excluding outliers. Where exclusion significantly improved the classification accuracy of the models (e.g., by more than 2%), these analyses were reported. Where classification accuracy was not significantly improved, then the baseline model was reported.

## 10. Results

Participants reported high rates of HED, with 332 (69.7%) engaging in at least one episode of heavy episodic drinking during the previous month. This sample also experienced significant rates of MBA involvement, confirming their high-risk status, with 148 (31.1%) and 160 (33.6%) citing physical and verbal MBA victimization, respectively, while 100 (21%) reported physical MBA perpetration and 115 (24.2%) reported perpetrating verbal MBA at least once during the past year. Means, standard deviations and scale reliabilities for the present study as well as correlations among variables are presented in Table 1. The Cronbach’s α for the multi-item measures indicated generally acceptable reliability for relevant scales [64].

Physical MBA perpetration was moderately positively correlated with verbal MBA perpetration (*r* = 0.52) and with both verbal and physical MBA victimization (*r* = 0.42 and *r* = 0.59, respectively). Physical victimization had a moderate to strong positive correlation with verbal victimization (*r* = 0.65). HED (Y/N) shared weak, but significant correlations with all four aggression outcomes as well as the Trait Physical and Trait Anger scales, impulsivity, male honour, CMNI Violence, CMNI Risk-taking and CMNI Playboy. The Trait Physical dimension had a moderately positive correlation with trait aggression’s other factors in Trait Verbal (*r* = 0.42), Trait Anger (*r* = 0.51) and Trait Hostility (*r* = 0.32). The Trait Physical aggression domain was also significantly correlated with impulsivity (*r* = 0.44) and CMNI Violence (*r* = 0.43).

Table 2, Table 3, Table 4 and Table 5 provide the logistic regression results including *Beta*-values, Wald statistics, odds ratios (OR) and the 95% confidence intervals for each step of the respective models.

### 10.1. Predictors of MBA Perpetration (Physical and Verbal)

In relation to physical MBA perpetration (see Table 2), the model at step 1 which included HED and key personality variables was significant, χ^2^ (7, *n* = 462) = 123.43, *p* < 0.001. HED (OR = 8.44), Trait Physical (OR = 3.05), Trait Anger (OR = 1.82) and narcissism (OR = 1.29) were all significantly and positively associated with physical MBA perpetration in the multivariate model, while Trait Hostility (OR = 0.48) significantly reduced the odds of reporting physical MBA perpetration. The addition of male honour and the masculine norms at step 2 significantly improved the model, χ^2^ (7, *n* = 462) = 44.66, *p* < 0.001. The full model was significant, χ^2^ (14, *n* = 462) = 168.09, *p* < 0.001. HED (OR = 10.35), Trait Physical (OR = 2.05), Trait Anger (OR = 2.16), narcissism (OR = 1.31) and CMNI Violence (OR = 7.73) all made unique and significant positive contributions to the final model while Trait Hostility (OR = 0.49) remained a protective factor with CMNI Playboy (OR = 0.37) also significantly reducing the odds of reporting physical MBA perpetration.

With respect to verbal MBA perpetration (see Table 3), the model including key personality variables and HED at step 1 was significant, χ^2^ (7, *n* = 464) = 90.40, *p* < 0.001. HED (OR = 6.02), Trait Physical (OR = 2.04) and narcissism (OR = 1.30) were significantly and positively associated with verbal MBA perpetration while Trait Hostility (OR = 0.54) significantly reduced the odds of such aggression. The inclusion of male honour and masculine norms significantly improved the model at step 2, χ^2^ (7, *n* = 464) = 23.49, *p* < 0.01. In the final model which was significant, χ^2^ (14, *n* = 464) = 113.89, *p* < 0.001, HED (OR = 6.73), Trait Physical (OR = 1.85), narcissism (OR = 1.36), CMNI Winning (OR = 2.15) and CMNI Violence (OR = 2.87) all significantly increased the odds of verbal MBA perpetration while Trait Hostility (OR = 0.56) remained significantly and negatively related.

In each of the models, Trait Verbal, impulsivity, male honour and masculine norms CMNI Emotional Control (restricted emotional expression), CMNI Risk-taking (willingness to take risks) and CMNI Heterosexual Self-presentation (HSP) were not significantly related to either physical or verbal barroom aggression perpetration.

### 10.2. Predictors of MBA Victimization (Physical and Verbal)

In relation to victimization from physical MBA (see Table 4), the model at step 1 including HED, trait aggression’s four-factors, impulsivity and narcissism was significant, χ^2^ (7, *n* = 468) = 95.66, *p* < 0.001, with HED (OR = 5.72), Trait Physical (OR = 1.54) and Trait verbal (OR = 1.41) serving as significant and positive predictors while Trait Hostility (OR = 0.69) significantly reduced the odds of citing victimization from physical MBA. The addition of male honour and masculine norms significantly improved the model at step 2, χ^2^ (7, *n* = 468) = 21.53, *p* < 0.01. The full model was significant, χ^2^ (14, *n* = 468) = 117.19, *p* < 0.001. At this step, Trait Physical, Trait Verbal and Trait Hostility no longer made unique and significant contributions to the model, with HED (OR = 6.23) having the only significant positive association while CMNI Playboy (desire for multiple sexual partners; OR = 0.55) and CMNI HSP (OR = 0.56) significantly decreased the odds of experiencing physical barroom aggression victimization.

With respect to verbal MBA victimization (see Table 5), at step 1, the model incorporating HED and personality-related variables was significant, χ^2^ (7, *n* = 476) = 44.64, *p* < 0.001, with HED (OR = 2.97) and Trait Physical (OR = 1.23) making unique and significant contributions. At step 2, the introduction of male honour and masculine norms significantly improved the model, χ^2^ (7, *n* = 476) = 19.22, *p* < 0.01. The full model with all variables was also significant, χ^2^ (14, *n* = 476) = 63.85, *p* < 0.001. At this step, Trait Physical was no longer a significant contributor, leaving HED (OR = 3.02), CMNI Winning (OR = 1.78) and male honour (OR = 1.39) as the only variables to significantly increase the risks of being a victim of such aggression with conformity to CMNI HSP (OR = 0.57) again significantly reducing the odds of reporting verbal MBA victimization.

Analyses of both MBA victimization models revealed no significant associations between Trait Anger, impulsivity, CMNI Violence, CMNI Risk-taking and CMNI Emotional Control and either verbal or physical barroom aggression victimization.

## 11. Discussion

Participants reported high levels of HED, with nearly 70% engaging in at least one session of heavy drinking during the previous month, which is higher than rates found in previous samples of Australian (47%) [12] and Canadian (<20%) [51] male university students. Reinforcing their high-risk status were significant rates of barroom aggression involvement with more than one-fifth (21%) citing physical perpetration and nearly one-quarter (24.2%) reporting verbal perpetration. Additionally, nearly one-third had experienced physical (31.1%) and verbal (33.6%) MBA victimization. These rates far exceed previous studies of samples drawn from those attending nightlife settings across Australia which found an average of 15% of participants reported aggression involvement, either as perpetrators or victims [2]. These comparisons indicate Australian construction workers experience significantly more aggression in or around licensed venues than most of the broader population and thus deserve more attention by alcohol and aggression researchers seeking to better understand the drivers of alcohol-related aggression generally and barroom aggression specifically.

### 11.1. Heavy Episodic Drinking and Male Barroom Aggression

HED was a significant contributor to aggression involvement across all four models, significantly increasing the risks of perpetration of and victimization from verbal and physical MBA. This accords with previous findings [29], and suggests that eight or more standard alcoholic drinks (80 g of alcohol) in a single session may have an effect on frontal lobe functioning among heavy drinking populations such as those used in the present study with the resultant modifications to neurochemical systems likely to lower inhibitions and the threshold for perpetrating aggression while also impacting on problem-solving abilities and the capacity to deal with threatening and or potentially volatile situations [65], which then heightens the risks of victimization.

### 11.2. Predictors of MBA Perpetration

Trait Physical aggression, which has been used previously as a proxy for general aggressiveness [31,66] was positively associated with verbal and physical MBA perpetration. This is consistent with prior research linking this subscale with verbally aggressive behaviour in naturalistic settings [33], reports of fights in licensed venues [33] and the severity of barroom aggression [36]. The associations with both verbal and physical MBA perpetration accords with postulations, that in public spaces such as bars, clubs or pubs, verbal aggression can escalate into physical violence [65].

Trait Anger significantly increased the odds of physical MBA perpetration which accords with prior research indicating this component can contribute to alcohol-related aggression (ARA) generally [34]. However, this study confirms a role in barroom aggression specifically, which reinforces suggestions that ARA may stem from the readiness with which those high on anger detect aggression, threats and dangers in their immediate environment [34], with such cues a common feature within bars, clubs or pubs. The addition of alcohol may further impair the regulation of behaviour given some have suggested trait aggressiveness is associated with poor neuro-psychological skills [6]. Indeed, the barroom environment may be particularly conducive to the expression of these individual differences given proscriptive norms about socially acceptable behaviour may be weaker in such settings [36,67] with alcohol use providing an additional ‘alibi’ to “let go” and act on innate tendencies [65].

Contrasting with predictions, Trait Hostility reduced the odds of verbal and physical MBA perpetration. Perhaps alcohol can lead to hostile feelings in some rather than overt acts of aggression [68], with those high in hostility using alcohol as a coping mechanism as opposed to a catalyst for aggressiveness. One possible explanation is that those who score higher on Trait Hostility, controlling for other scales on the AQ, may prefer avoidant coping strategies rather than confrontational strategies in provoking situations [69], a pattern that may preclude construction workers high in hostility from initiating aggression and or responding to the provocative behaviour of others in a bar setting. This assertion is consistent with findings that high hostility is associated with a lack of confidence in resolving aggressive situations satisfactorily following provocation [70], tendencies which may reduce the risks of MBA perpetration.

Impulsivity was unrelated to MBA perpetration which accords with some prior research indicating it was not a predictor to the occurrence of barroom aggression [40]. Some evidence suggests that impulsivity in isolation does not relate directly to aggression involvement, but rather exerts an influence on poor social problem-solving abilities [71]. Impulsive individuals in the present sample may have appropriate social problem-solving skills that temper any involvement in MBA. Others have also noted the importance of ‘urgency’ in relation to impulsivity which reflects the tendency to act rashly and engage in regrettable actions in response to positive or negative mood states [37]. Perhaps, whether or not an impulsive male will aggress in a barroom setting is dependent on his current mood state, a confounder not directly assessed in the current study which may have obscured the strength of any associations with MBA perpetration.

Interestingly, the present study establishes narcissism as a risk factor for both verbal and physical MBA perpetration, which is consistent with previous research that narcissism has been associated with problematic alcohol use [17] and aggression, particularly in response to provocation or ‘ego threat’ [43,44]. There are a number of possible explanations. By nature many narcissists have fragile self-views which can be easily punctured, a tendency which may be especially relevant in bars, clubs or pubs given the tendency for strangers to make superficial judgements which can pose a threat to inflated self-concepts, particularly if narcissistic individuals are using alcohol to cope with associated negative affect [17]. These superficial judgements which can often contradict or challenge favourable self-views, in conjunction with the use of alcohol may induce a narcissist to aggress against the source(s) of that threat, leaving them vulnerable to provocation which is only emphasised further by a general lack of empathy and sensitivity to personal insults [45]. Further, the present sample may include more ‘overt’ than ‘covert’ narcissists, with such individuals often experiencing greater interpersonal disruption given a propensity for unpredictability and acting out unexpectedly [72] which may manifest into aggressive behaviour in or around public drinking establishments.

Of the masculinity-related factors, CMNI Violence increased the odds of reporting verbal and physical MBA perpetration while CMNI Winning was a positive predictor of verbal MBA perpetration only. At face value, this suggests that construction workers endorsing the need for violence and with a drive to win are most likely to perpetrate barroom aggression, a setting proposed as a site renowned for the competitive performance of masculinity [73]. The present findings with respect to CMNI Winning are consistent with elements of research undertaken by Miller et al. [9] who reported this domain was a significant predictor of barroom aggression among a high-risk and related sample of Australian tradesmen; however, unlike the work of Miller and colleagues [9], the current study found that CMNI Violence significantly associated with verbal and physical MBA perpetration. This finding does, however, accord with Wells et al. [51]. The lack of any positive associations between masculine norms other than CMNI Winning and CMNI Violence may suggest that they play either no or only a peripheral role in explaining MBA, particularly comparative to aspects of personality. Indeed, CMNI Winning and CMNI Violence may share similar features to trait-like individual-difference variables synonymous with dispositional or personality-based frameworks. CMNI Winning may represent a competitive nature while CMNI Violence may be related more to a dispositional style than a prescribed norm recognized and endorsed as a key component to one’s own or others’ masculinity. Indeed, CMNI violence had a robust correlation with the Trait Physical subscale, which can be posited as the most striking representation of an aggressive personality (*r* = 0.43). The potential overlap between some norms and trait variables has been noted by others, particularly in relation to aggression [74] with concerns about the need for greater clarity regarding the incremental validity of certain masculinity-related measures over and above trait variables [74]. The issue being any attempts to elucidate the role of gender-linked variables in predicting important outcomes such as aggression perpetration may be consistently complicated by issues of shared variance with individual differences in interpersonal styles as captured by trait aggressiveness, trait agreeableness and or other associated aspects of personality [74].

CMNI Playboy was a significant factor in the final model for physical MBA perpetration by reducing the odds of any such involvement. While this is inconsistent with several previous studies [9,51], it does make intuitive sense. Construction workers adhering to this norm associated with a desire for multiple sexual partners are likely to prioritise such pursuits over and above any interest in perpetrating physical aggression against other males in a barroom context given that it may be an unwanted and unnecessary distraction from the search for sexual encounters while also posing a threat to ego and image concerns, the protection of which takes priority.

### 11.3. Predictors of MBA Victimization

At step 1, prior to the introduction of masculinity-related factors, Trait Physical aggression was a positive predictor of both physical and verbal MBA victimization while Trait Verbal was associated with an increased risk of physical victimization. These findings accord with prior research indicating men’s perpetration of aggression is also associated with reports of victimization [75] given the tendency for some aggressive males to cite experiences of aggression were mutual or instigated by another [76], thus making them victims. While on the surface, this may blur the lines between perpetration and victimization, it may also accord with, and lend weight to, the contention that the personality characteristics of opponents jointly influence the escalation of aggression, especially in drinking contexts [40], and that in a barroom context, perpetrators and victims may share similar dispositions. This study is among the first to provide some empirical support for such an assertion.

Interestingly, Trait Hostility again protected participants against MBA, reducing the odds of reporting physical victimization at step 1. While this appears counter-intuitive, it may be that among the current sample whose members work in high-risk and high-stress environments where alienation and powerlessness are factors [77], innate hostility may inspire a degree of rumination, and rather than rumination on ‘anger’, construction workers may ruminate on ‘sadness’, in which case the introduction of alcohol at bars, clubs or pubs is likely to reduce aggression rather than augment it [78]. Rather than using alcohol to fuel aggression, construction workers with high hostility, like many other men, may drink to escape, deal with ongoing distress and relieve accompanying depression [79].

It is worth noting, however, that the respective aspects of trait aggression were rendered non-significant in the final models incorporating all variables, both in relation to verbal and physical victimization. It may reinforce suggestions of shared variance between components of trait variables and masculinity-related factors or alternatively that elements of personality which drive an aggressive disposition are more profound in the prediction of perpetration than victimization.

Male honour was significantly and positively related to verbal MBA victimization, while CMNI Heterosexual Self Presentation was negatively related to both verbal and physical MBA victimization, and CMNI Playboy was negatively related to physical MBA victimization. Given the number of analyses and inter-correlations among variables, these may be chance findings but they may also suggest interesting directions for future research. Given face-saving and impression management are ongoing concerns for many in bars, clubs or pubs, individuals for whom male honour is an issue may be acutely sensitive to verbal slights or insults, making them highly salient which improves recall of any such victimization. Further, such concerns can often result in retaliatory aggression as opposed to its initiation [80], which may help explain male honour’s association with aspects of MBA victimization.

CMNI Winning was significantly and positively associated with verbal MBA victimization. The verbal sparring and brinkmanship so often experienced in a barroom context may provide an outlet for those with a competitive nature and or who view winning as an essential feature of the masculine with such individuals viewing it as an acceptable risk, unlike, physical violence where the consequences are more serious and the outcomes uncertain, limiting one’s confidence they will emerge a winner. Such considerations may therefore increase the risks of verbal MBA involvement but temper any associations with physical MBA, whether as perpetrators or victims.

CMNI Playboy reduced the odds of reporting physical MBA victimization and similar explanations to those outlined earlier in relation to perpetration may apply, namely those for whom a pre-occupation with meeting and or interacting with potential sexual partners exists, there may be a lack of interest in engaging in MBA given any such involvement can be counter-productive to the primary purpose for attending licensed venues. In essence, those committed to finding a prospective sexual partner may devote little time and attention to engaging with other males, lessening the likelihood of involvement in MBA. Further, it is possible that this particular subscale represents, in part, a participant’s relationship status in that those endorsing a desire for multiple sexual partners may be more likely to be single, which as a consequence lessens the likelihood those who are heterosexual will encounter situations where they feel compelled to defend a female partner from the unwanted and or provocative approaches of other males in public drinking contexts with such behaviours a common source of angst and or conflict. The absence of or reduced exposure to such scenarios among those who are single and who, by extension may be more likely to endorse a desire to be a playboy or promiscuous, may significantly protect them against many of the situations which so often inspire episodes of MBA victimization.

CMNI Heterosexual Self-presentation also significantly protected construction workers against MBA victimization, both verbal and physical. While others have linked this norm to reduced MBA perpetration [12], this study demonstrated an association with victimization. There are a number of possible explanations, namely males endorsing this norm which reflects a profound concern for how they are perceived by others in relation to heterosexuality/homosexuality may be hyper-vigilant with respect to self-monitoring in licensed venues, reducing any attention-drawing behaviour which may place them at risk of experiencing MBA or, alternatively, those individuals consumed by a dread for appearing homosexual may develop a hyper-masculine façade [12], protecting them against victimization in bars, clubs or pubs.

## 12. Limitations and Future Research

This study relied on self-reports, leaving it open to ‘social desirability’ responding. Additionally, some of the inconsistencies which emerged in relation to trait aggression’s four factors may support the view that dispositional aggressiveness is best thought of as a unitary construct [25]. Given the present study was cross-sectional, causal inferences cannot be drawn between predictor variables and MBA perpetration and victimization. Additionally, the purposive sample of high-risk construction workers limits generalizability to other populations, particularly those at low-risk of MBA exposure. The retrospective nature of the measures used in the present study may have increased recall error, resulting in under-reporting on key variables such as HED and aggression involvement. The Impulsivity scale did not assess the role of ‘urgency’ (the tendency to act rashly and engage in regrettable actions in response to positive or negative mood states), which may have obscured this construct’s relationships with MBA, and narcissism was examined using a single-item, a decision made in the interests of brevity. While the scale developers produced evidence across a number of studies that demonstrated its discriminant, convergent and predictive validity as well as suitable test–retest reliability [44], future research wishing to replicate or extend on the present findings may benefit from using more comprehensive measures of narcissism and impulsivity.

## 13. Conclusions

This sample of Australian construction workers confirmed their at-risk status for MBA, reporting high levels of both HED and MBA comparative to other samples used in much of the previous related research. These findings reinforce the need to investigate the utility of key variables among populations where exposure to barroom aggression is most prevalent. Although personality factors were significantly related to most measures of MBA, HED emerged as the most significant and consistent predictor of aggression involvement which highlights the importance of alcohol in contributing to aggressive behaviour. A core strength of the present study is the investigation of the relationships between these personality traits, normative attitudes and alcohol consumption variables and the prediction of victimization from verbal and physical MBA. This provides early insights into a potential profile of those at risk of aggression at bars, clubs or pubs, which might assist in building appropriate interventions designed to reduce incidents of what has become a significant public health and policing issue. Our findings suggest that personality (especially those aspects related to a history of physical aggression, trait anger and narcissism) is relevant in the prediction of MBA perpetration over and above most of the masculinity-related factors examined. This casts a shadow over the predictive utility of existing measures of conformity to masculine norms in an Australian context given they were derived from and designed for use in North American populations (mostly university students), and thus may not represent norms recognized or endorsed by others.

## Figures and Tables

**Table 1:** Descriptive Statistics, Scale Reliabilities and Pearson Correlation Coefficients for Key Variables.

	1	2	3	4	5	6	7	8	9	10	11	12	13	14	15	16	17	18
MBA Victimization																		
1. Physical	1	0.65 **	0.59 **	0.47 **	0.25 **	0.25 **	0.23 **	0.22 **	0.05	0.15 **	0.21 **	0.13 **	0.18 **	0.08	0.17 **	0.00	−0.08	0.16 **
2. Verbal		1	0.42 **	0.58 **	0.23 **	0.19 **	0.16 **	0.13 **	0.03	0.08	0.16 **	0.15 **	0.13 **	0.07	0.14 **	0.03	−0.09	0.17 **
MBA Perpetration																		
3. Physical			1	0.52 **	0.22 **	0.32 **	0.22 **	0.24 **	0.04	0.18 **	0.20 **	0.12 **	0.30 **	0.10 *	0.19 **	−0.00	−0.05	0.20 **
4. Verbal				1	0.19 **	0.24 **	0.19 **	0.13 **	0.01	0.15 **	0.19 **	0.15 **	0.22 **	−0.01	0.13 **	0.05	0.00	0.09 *
5. HED					1	0.14 **	0.07	0.11 *	0.05	0.04	0.13 **	−0.02	0.11 *	0.04	0.15 **	0.18 **	−0.07	0.11 *
Traits																		
6. TPhysical						1	0.42 **	0.51 **	0.32 **	0.19 **	0.44 **	0.12 *	0.43 **	0.08	0.35 **	0.17 **	0.12 **	0.36 **
7. TVerbal							1	0.52 **	0.30 **	0.29 **	0.41 **	0.27 **	0.19 **	0.04	0.21 **	0.11 *	0.09	0.26 **
8. TAnger								1	0.42 **	0.20 **	0.34 **	0.11 *	0.20 **	0.07	0.16 **	0.13 **	0.09	0.25 **
9. THostility									1	0.15 **	0.23 **	0.05	0.07	0.05	0.00	0.02	0.16 **	0.21 **
10. Narcissism										1	0.15 **	0.05	0.07	0.05	0.07	0.04	0.02	0.10 *
11. Impulsivity											1	0.19 **	0.19 **	0.14 **	0.28 **	0.17 **	0.05	0.21 **
Masculinity/CMNI																		
12. Winning												1	0.17 **	0.11 *	0.22 **	0.12 **	0.08	0.13 **
13. Violence													1	0.08	0.29 **	0.21 **	0.06	0.26 **
14. Emotional Control														1	0.03	0.07	0.06	0.17 **
15. Risk-Taking															1	0.19 **	0.08	0.26 **
16. Playboy																1	0.07	0.15 **
17. HSP																	1	0.21 **
18. Male Honour																		1
Mean						2.78	2.83	2.70	2.55	2.62	2.14	2.59	2.51	2.51	2.82	2.33	2.26	2.28
SD						0.87	0.82	0.83	0.75	1.43	0.85	0.47	0.51	0.57	0.48	0.52	0.52	0.82
Scale Reliabilities						0.71	0.72	0.73	0.72	-	0.87	0.61	0.69	0.86	0.81	0.71	0.71	0.60

Scale Ranges: 1–5 (Trait Physical, Verbal, Anger, Hostility), 1–7 (Narcissism), 1–4 (Impulsivity), 1–5 (Male Honour), 1–4 (Masculine Norms), HED (Y/N) = Heavy Episodic Drinking (at least one episode in past month). * *p* < 0.05, ** *p* < 0.01, two-tailed. TPhysical = Trait Physical, TVerbal = Trait Verbal, TAnger = Trait Anger, THostility = Trait Hostility. HSP = Heterosexual Self-presentation.

**Table 2:** Hierarchical Binary Logistic Regression Predicting Physical MBA Perpetration.

		Step 1				Step 2		
	*B* (SE)	Wald χ^2^	OR	95% CI	*B* (SE)	Wald χ^2^	OR	95% CI
HED	2.13(0.48)	19.92	8.44 ***	3.31–21.55	2.34(0.54)	18.74	10.35 ***	3.59–29.81
Trait Physical	1.12(0.23)	24.48	3.05 ***	1.96–4.74	0.72(0.26)	7.91	2.05 **	1.24–3.39
Trait Verbal	0.19(0.21)	0.82	1.20	0.81–1.80	−0.05(0.23)	0.04	0.95	0.60–1.50
Trait Anger	0.60(0.22)	7.75	1.82 **	1.19–2.77	0.77(0.24)	10.50	2.16 **	1.36–3.44
Trait Hostility	−0.74(0.21)	12.06	0.48 **	0.32–0.73	−0.72(0.24)	9.25	0.49 **	0.31–0.77
Narcissism	0.25(0.10)	6.28	1.29 *	1.06–1.56	0.27(0.11)	6.05	1.31 *	1.06–1.63
Impulsivity	0.11(0.18)	0.36	1.12	0.78–1.60	0.09(0.22)	0.18	1.09	0.78–1.67
CMNI Winning					0.44(0.35)	1.59	1.55	0.78–3.07
CMNI Violence					2.05(0.43)	22.63	7.73 ***	3.33–17.95
CMNI Emotional Control					0.28(0.28)	1.00	1.32	0.77–2.28
CMNI Risk-Taking					0.22(0.43)	0.27	1.25	0.54–2.87
CMNI Playboy					−0.99(0.33)	8.97	0.37 **	0.19–0.71
CMNI Heterosexual Self-Presentation					−0.38(0.31)	1.55	0.68	0.38–1.24
Male Honour					0.29(0.20)	1.98	1.33	0.89–1.99

Step 1: Hosmer and Lemeshow R^2^ = 0.276. Step 2: Hosmer and Lemeshow R^2^ = 0.376. * *p* < 0.05, ** *p* < 0.01, *** *p* < 0.001. HED = Heavy Episodic Drinking; *B* = beta values, (SE) = standard error, OR = odds ratio, and CI = confidence interval.

**Table 3:** Hierarchical Binary Logistic Regression Predicting Verbal MBA Perpetration.

		Step 1				Step 2		
	*B* (SE)	Wald χ^2^	OR	95% CI	*B* (SE)	Wald χ^2^	OR	95% CI
HED	1.79(0.39)	20.87	6.02 ***	2.79–12.99	1.91(0.41)	21.71	6.73 ***	3.02–15.02
Trait Physical	0.72(0.19)	14.76	2.04 ***	1.42–2.94	0.61(0.21)	8.31	1.85 **	1.22–2.81
Trait Verbal	0.24(0.18)	1.69	1.27	0.89–1.82	0.06(0.20)	0.09	1.06	0.72–1.56
Trait Anger	0.03(0.20)	0.03	1.03	0.71–1.51	0.08(0.20)	0.15	1.08	0.73–1.61
Trait Hostility	−0.61(0.19)	10.40	0.54 **	0.37–0.79	−0.59(0.20)	8.70	0.56 **	0.38–0.82
Narcissism	0.26(0.09)	8.76	1.30 **	1.09–1.55	0.31(0.09)	10.98	1.36 **	1.13–1.64
Impulsivity	0.28(0.16)	2.94	1.32	0.96–1.82	0.33(0.18)	3.49	1.39	0.98–1.96
CMNI Winning					0.77(0.29)	6.88	2.15 **	1.21–3.81
CMNI Violence					1.05(0.32)	10.62	2.87 **	1.52–5.39
CMNI Emotional Control					−0.40(0.23)	2.89	0.67	0.42–1.06
CMNI Risk-Taking					−0.18(0.33)	0.31	0.83	0.44–1.59
CMNI Playboy					−0.38(0.26)	2.14	0.68	0.41–1.14
CMNI Heterosexual Self-Presentation					−0.02(0.26)	0.01	0.98	0.59–1.63
Male Honour					−0.13(0.18)	0.57	0.88	0.62–1.24

Step 1: Hosmer and Lemeshow R^2^ = 0.183. Step 2: Hosmer and Lemeshow R^2^ = 0.231. * *p* < 0.05, ** *p* < 0.01, *** *p* < 0.001. HED = Heavy Episodic Drinking; *B* = beta values, (SE) = standard error, OR = odds ratio, and CI = confidence interval.

**Table 4:** Hierarchical Binary Logistic Regression Predicting Physical MBA Victimization.

		Step 1				Step 2		
	*B* (SE)	Wald χ^2^	OR	95% CI	*B* (SE)	Wald χ^2^	OR	95% CI
HED	1.74(0.33)	28.41	5.72 ***	3.01−10.90	1.83(0.34)	28.54	6.23 ***	3.19–12.20
Trait Physical	0.43(0.16)	7.10	1.54 **	1.12–2.11	0.34(0.19)	3.42	1.41	0.98–2.03
Trait Verbal	0.34(0.17)	4.20	1.41 *	1.02–1.96	0.26(0.18)	2.09	1.29	0.91–1.83
Trait Anger	0.26(0.18)	2.19	1.30	0.92–1.83	0.29(0.18)	2.60	1.34	0.94–1.91
Trait Hostility	−0.37(0.17)	4.74	0.69 *	0.50–0.96	−0.32(0.18)	3.22	0.73	0.51–1.03
Narcissism	0.15(0.08)	3.27	1.16	0.99–1.36	0.15(0.09)	3.22	1.16	0.99–1.37
Impulsivity	0.20(0.15)	1.79	1.22	0.91–1.63	0.17(0.16)	1.15	1.19	0.87–1.62
CMNI Winning					0.50(0.27)	3.50	1.65	0.98–2.80
CMNI Violence					0.40(0.27)	2.19	1.49	0.88–2.52
CMNI Emotional Control					0.27(0.21)	1.68	1.31	0.87–1.98
CMNI Risk-Taking					0.20(.29)	0.49	1.23	0.69–2.16
CMNI Playboy					−0.60(0.24)	6.07	0.55 *	0.34–0.89
CMNI Heterosexual Self-Presentation					−0.58(0.24)	5.83	0.56 *	0.35–0.90
Male Honour					0.12(0.16)	0.56	1.13	0.83–1.54

Step 1: Hosmer and Lemeshow R^2^ = 0.167. Step 2: Hosmer and Lemeshow R^2^ = 0.205. * *p* < 0.05, ** *p* < 0.01, *** *p* < 0.001. HED = Heavy Episodic Drinking, *B* = beta values, (SE) = standard error, OR = odds ratio, and CI = confidence interval.

**Table 5:** Hierarchical Binary Logistic Regression Predicting Verbal MBA Victimization.

		Step 1				Step 2		
	*B* (SE)	Wald χ^2^	OR	95% CI	*B* (SE)	Wald χ^2^	OR	95% CI
HED	1.09(0.25)	18.67	2.97 ***	1.81–4.86	1.11(0.26)	17.83	3.02 ***	1.81–5.05
Trait Physical	0.28(0.15)	3.83	1.23 *	1.00–1.77	0.23(0.17)	1.97	1.26	0.91–1.74
Trait Verbal	0.21(0.15)	1.83	1.23	0.91–1.67	0.11(0.16)	0.42	1.11	0.81–1.53
Trait Anger	0.44(0.16)	0.07	1.05	0.76–1.43	0.06(0.16)	0.12	1.06	0.77–1.46
Trait Hostility	−0.17(0.15)	1.24	0.85	0.63–1.14	−0.14(0.07)	0.83	0.87	0.64–1.18
Narcissism	0.04(0.07)	0.36	1.05	0.90–1.21	0.05(0.08)	0.40	1.05	0.90–1.22
Impulsivity	0.14(0.14)	1.01	0.31	0.88–1.50	0.10(0.14)	0.46	1.10	0.83–1.46
CMNI Winning					0.57(0.24)	5.73	1.78 *	1.11–2.84
CMNI Violence					0.05(0.24)	0.46	1.05	0.66–1.67
CMNI Emotional Control					0.08(0.19)	0.20	1.09	0.75–1.57
CMNI Risk-Taking					0.07(0.25)	0.07	1.07	0.65–1.75
CMNI Playboy					−0.26(0.21)	1.54	0.77	0.51–1.17
CMNI Heterosexual Self-Presentation					−0.56(0.22)	6.71	0.57 **	0.37–0.87
Male Honour					0.33(0.14)	5.24	1.39 *	1.05–1.84

Step 1: Hosmer and Lemeshow R^2^ = 0.073. Step 2: Hosmer and Lemeshow R^2^ = 0.105. * *p* < 0.05, ** *p* < 0.01, *** *p* <0.001. HED = heavy episodic drinking. *B* = beta values, (SE) = standard error, OR = odds ratio, and CI = confidence interval.

## Data Availability

Data can be made available subject to ethical approval.
